# Self-Determination and Goal Orientation in Track and Field

**DOI:** 10.2478/v10078-012-0054-0

**Published:** 2012-07-04

**Authors:** Ngien-Siong Chin, Selina Khoo, Wah-Yun Low

**Affiliations:** 1 Physical Education and Health Department, Tun Abdul Razak Teachers’ Education Institute, Kota Samarahan, MALAYSIA.; 2 Sports Centre, University of Malaya, Kuala Lumpur, MALAYSIA.; 3 Faculty of Medicine, University of Malaya, Kuala Lumpur, MALAYSIA.

**Keywords:** adolescent athletes, achievement goals, motivation, gender, age, locality

## Abstract

This study investigated gender, age group and locality differences in adolescent athletes’ self-determination motivation and goal orientations in track and field. It also examined the relationship between the self-determination theory and achievement goal theory. A total of 632 (349 boys, 283 girls) adolescent athletes (aged 13–18 years) completed the Sports Motivation Scale and Task and Ego Orientation in Sport Questionnaire. Results indicated significant differences between gender on intrinsic motivation, extrinsic motivation, amotivation (t(630) = 4.10, p < 0.05) and ego orientation (t(630) = 2.48, p < 0.05). Male students reported higher intrinsic motivation, extrinsic motivation, amotivation and ego orientation. A significant difference was found between age groups on task orientation (t(630) = 1.94, p < 0.05) and locality on ego orientation (t(630) = 1.94, p < 0.05). Older athletes showed significantly higher task orientation. Rural athletes had higher ego orientation whereas urban athletes have higher intrinsic motivation. Task orientation was related to intrinsic motivation (r = 0.55, p < 0.01), extrinsic motivation (r = 0.55, p < 0.01), but weakly related to amotivation (r = 0.10, p < 0.01). Ego orientation was related to intrinsic motivation (r = 0.30, p < 0.01), extrinsic motivation (r = 0.36, p < 0.01) and amotivaion (r = 0.36, p < 0.01). Task orientation was related to ego orientation (r = 0.29, p < 0.01). Multiple regression analysis showed intrinsic motivation, extrinsic motivation and amotivation accounted for 30.5% of the variances in task orientation.

## Introduction

Motivation plays a vital role in sports as it influences why and how athletes engage in the activities they choose, affecting the quality of their engagement and ultimately the outcome of their effort. Research on motivation in sports and physical education has utilized the self-determination theory and achievement goal theory to predict the motivation of athletes.

The self-determination theory (Deci and Ryan, 1985) is a contemporary framework that is increasingly used to understand motivation in the sports and physical education domains. According to the theory, the different motivational orientations serve as stimuli to act. Motivational orientations differ in the extent to which they are self-determined, or emanate from one’s personal interests and values (Ryan and Deci, 2000). The self-determination theory comprises of motivation and the basic needs for autonomy, relatedness and competence. The self-determination theory categorizes motivation into three types namely intrinsic motivation, extrinsic motivation, and amotivation to account for the different reasons why individuals engage in activities (Deci and Ryan, 1985). Intrinsic motivation means engaging in activities for their own sake, namely for the feelings of pleasure, interest, and satisfaction that is directly derived from participation. When intrinsically motivated, individuals are fully self-regulated, engage in activities out of interest, experience a sense of volition, and function without the aid of external rewards and/or constraints (Deci and Ryan, 1985). Extrinsic motivation refers to a situation where an individual engages in an activity for an instrumental purpose as a means to an end and not for its own sake. For example, athletes who participate in athletics because they are adhering to their parents’ wishes are participating through extrinsic reasons. Amotivation is perceptions of incompetence, lack of control and non-intended action (Ryan and Deci, 2000). Throughout the self-determination literature, amotivation is viewed as developmentally disruptive, because it is linked with low levels of need satisfaction, less perceived control, and negatively associated with overall well-being (Ryan and Deci, 2000). According to Ryan and Deci (2000), humans have basic needs for autonomy, competence and relatedness. Autonomy is defined as freedom of choice. The need for relatedness is the need to feel a sense of belonging and connectedness with others. The need for competence is met when one feels capable, such as when receiving positive and informational feedback. When those needs are satisfied, individuals are motivated, productive and more self-determined.

Achievement goal theory typically differentiates between two types of goal orientations: task and ego. Task orientation is related to developing competence by improving upon one’s skills, personal competence and task mastery. It is assumed that task orientation will lead to positive and adaptive achievement behaviors (Duda et al., 1995). Athletes with a task goal orientation tend to select and persist at challenging tasks because they value effort as a way to attain new skills. In contrast, ego orientation is based on one’s subjective evaluation of performance compared with that of others (Nicholls, 1989). Generally, ego orientation is associated with maladaptive motivational patterns that are dependent on an individual’s perceived ability (Xiang et al., 2004). Athletes who endorse an ego orientation tend to select tasks that are easier and tasks at which they perceive their chances of success will be high (Tyson et al., 2009).

Research has shown a link between these two theories that are concerned with the underlying motivations for an individual’s behavior though focusing on different dimensions of motivation. An ego orientation represents an internally controlling state that can undermine intrinsic motivation, whereas a task goal orientation represents a state in which individuals derives pleasure from participation that facilitates intrinsic motivation (Cox, 2002; Deci and Ryan, 1985). Task orientation predicted intrinsic motivation, but did not predict amotivation (Ntoumanis, 2001). Conversely, ego orientation was associated with extrinsic motivation. These studies show that task goal orientation fostered intrinsic motivation, whereas ego orientation promoted extrinsic motivation.

Among the factors that influence athletes’ perceptions of self-determination and goal orientations are socio-demographic characteristics like gender, age and locality.

### Gender differences

Adolescents’ self-determination of activities tends to differ mainly in sex stereotypic ways where females have higher self-determined motivational profiles than males in a diversity of sporting activities (Medic et al., 2007; Recours et al., 2004). Researchers have found that females tend to be more intrinsically motivated, whereas males tend to be more extrinsically-motivated in the sports context (Beaudoin, 2006). Intrinsically-motivated athletes participate more for pleasure, fun and satisfaction. In contrast, extrinsically-motivated athletes participate more for competition and the satisfaction of winning (Hellandsig, 1998). Other studies have shown that extrinsically-motivated male athletes tend to focus on rewards and recognition whereas intrinsically-motivated female athletes focus more on fun and task mastery (Tuffey, 2000).

Researchers have also found that females tend to be more task-oriented, whereas males tend to be more ego-oriented in the sports context (Li et al., 1996). For example, in the study by (White et al., 1998), task-oriented athletes tended to believe that sports would enhance cooperative skills, personal mastery, togetherness, and higher levels of enjoyment. In contrast, ego orientation was positively linked with the belief that sports would increase career mobility, enhance one’s popularity and, social status, and build a competitive spirit that tend to be associated with a lower levels of motivation (Duda, 1989). Other studies have found that ego oriented athletes adopt a normative conception of ability leading them to conclude that winning and beating others are their main priorities (Duda, 1989; Duda et al., 1995; Pensgaard and Roberts, 2003). However, Omar-Fauzee et al., (2008) found no differences between male and female athletes in goal orientations as athletes had both high task and ego orientations.

### Age differences

Although many studies have been conducted on motivation, there has been very little research examining age-related differences on the types of motivation and goal orientations that leads to adolescent athletes’ participation in sports. It is expected that age could have some form of impact on types of motivation. Studies on age and motivation show different types of motivation among younger and older students. Biddle et al., (1999) as well as Digelidis and Papaioannou (1999) found that younger students had higher intrinsic motivation than older students participating in physical education. However, Tuffey (2000) found younger athletes to be more extrinsically-motivated than older ones, whereas older athletes showed greater amotivation. Many studies have shown that younger athletes tend to be more task-oriented than older athletes (Digelidis and Papaioannou, 1999; Weiss and Ferrer-Caja, 2002; Xiang and Lee, 2002; Xiang et al., 2004).

However, other studies (Christodoulidis et al., 2001; Tzetzis et al., 2002) found no age differences in task and ego orientation.

### Locality Differences

Few empirical studies have been conducted to examine differences in motivation between urban and rural youth in sports utilizing self-determination theory and achievement goal theory. A study by McHale et al. (2005) on sports involvement and urban school children showed that sports involvement had a positive influence on self-esteem and social competence. Many rural schools often face serious economic and resource constraints due to their remoteness, being socioeconomically disadvantaged, with limited facilities, funding and opportunities that place rural athletes at risk for low motivation and lack of success (Hardré et al., 2007). However, Freeman and Anderman (2005) found that rural students were more task-oriented than urban students due to mastery goal structures in their schools.

### Purpose of the study

The purpose of this study was threefold. First, to examine the differences in the types of motivation in terms of gender, age and locality. Second, to examine the differences in achievement goal orientations in terms of gender, age group and locality. Third, to examine the relationship between the self-determination theory and achievement goal theory.

## Material and Methods

### Participants and Procedures

The sample comprised 632 (349 males and 283 females) adolescent athletes who participated in the 34^th^ state level Sarawak School Sports Interdivision Athletic Meet in 2006. Sarawak is the largest state in Malaysia with 11 administrative divisions. These athletes represented their respective divisions in the competition and are considered the best in the state for the under-15 and under-18 age groups. The questionnaires were administered with the help of the team managers and coaches. This study was approved by the university committee. Permission for the study was granted by the Sarawak Education Department, divisional education officers, the Sarawak State Sports Council, team managers and coaches. Participation in the study was voluntary.

### Measures

This study used questionnaires that assessed the intrinsic motivation, extrinsic motivation, amotivation and achievement goals of adolescent athletes. The instrument was made up of three parts. The first part asked for the demographic information related to gender, age, locality and school. The second part measured intrisnsic motivation, extrinsic motivation and amotivation utilizing the Sport Motivation Scale (SMS) (Pelletier et al., 1995). The SMS was reworded to reflect track and field rather than the academic and physical education achievement domain. The third part measured the participant’s goal orientations which were assessed using the Task and Ego Orientation in Sport Questionnaire (TEOSQ) (Duda, 1989).

The 28-item SMS is based on self-determination theory and was designed to assess contextual intrinsic motivation, extrinsic motivation, and amotivation. Athletes responded to the item “Why do you practice your sport?” with responses from a Likert-type scale that ranges from 1 (does not correspond at all) to 7 (corresponds exactly). The SMS consists of seven subscales with four items attached to each.

The SMS showed good validity and reliability in sports and physical education settings (Alexandris et al., 2002; Pelletier et al., 1995). The SMS internal consistency values were 0.92 for intrinsic motivation, 0.84 for extrinsic motivation and 0.82 for amotivation (Alexandris et al., 2002).

The TEOSQ is a 13-item questionnaire that measures task orientation (7 items) and ego orientation (6 items). Participants were asked to think when they felt most successful in their sport. For the purpose of this study, the stem for all items was modified to, “I feel most successful in track and field when…”. Examples of items are: “…I work really hard” (task orientation) and “…others can’t do as well as me” (ego orientation). Responses are rated on a 5-point Likert scale from 1 (strongly disagree) to 5 (strongly agree). A mean score is calculated for both the task and ego subscales by adding the scores for each item on that sub-scale and divided by the number of items in that subscale. The mean score would range between 1 (low) and 5 (high) for each orientation.

The task and ego goal orientations also showed excellent reliability in the sports domains (Xiang and Lee, 2002; Xiang et al., 2004). In Malaysia, the TEOSQ has been translated into Bahasa Malaysia (the official language) and validated (Omar-Fauzee et al., 2008). The task and ego orientation subscales demonstrated adequate internal consistency with alpha reliability coefficients of 0.82 and 0.71, respectively.

A pilot test was carried out to investigate the reliability of the SMS and TEOSQ in Bahasa Malaysia. The pilot test was assessed using the test–retest reliability method on 36 athletes who completed the questionnaire over a 1-week interval. Results showed that the measures were stable.

### Analysis

The data was coded, edited and analysed using SPSS. Independent t-tests were conducted to examine the differences between gender, age group, and locality on intrinsic motivation, extrinsic motivation, amotivation and task and ego goal orientations. Person correlation was computed to identify relationships between achievement goal orientations, intrinsic motivation, extrinsic motivation and amotivation.

## Results

All the scales showed high reliability. Cronbach’s alpha coefficients for the full SMS, and the intrinsic motivation, extrinsic motivation and amotivation subscales were 0.91, 0.86, 0.84 and 0.53 respectively. The amotivation subscale was retained due to the theoretical relevance and importance to the research questions as well as substantiate from previous studies. The internal consistency coefficients for the full TEOSQ and the task and ego orientation subscales were 0.78, 0.73 and 0.78.

### Sociodemographic characteristics of participants

Out of the total number of participants who volunteered in the study (*n* = 632), 55.2% were male and 44.8% were female. The athletes’ mean age was 15.1 ± 1.2 years. The age-group categories showed that 66.6% of the respondents were 13–15 years old, whereas 33.4% were 16–18 years old.

Sarawak is the most multi-racial state in Malaysia with 27 ethnic groups. The distribution of participants in this study in terms of ethnicity showed that the Iban formed the largest percentage with 52.5%. This was followed by Chinese and Malay who comprised 16.9% and 11.6% of the population, respectively. The Bidayuhs and Indians formed the minority of the groups with only 5.7% and 0.3%, respectively. A total of 471 (74.5%) athletes were from rural areas and 161 (25.5%) athletes lived in urban locations.

Table 1 shows the independent t-test for intrinsic motivation, extrinsic motivation, amotivtion, task and ego goal orientations by sex, age group and locality. Male athletes (5.01 ± 0.87) reported significantly higher intrinsic motivation (*t* (630) = 4.10, *p* < 0.05) than female athletes (4.72 ± 0.92). Urban athletes (5.05 ± 0.94) reported significantly higher intrinsic motivation (*t* (630) = 2.65, *p* < 0.05) than rural athletes (4.83 ± 0.89). Male athletes (4.82 ± 0.89) reported significantly higher extrinsic motivation (*t* (630) = 4.10, *p* < 0.05) than female athletes (4.32 ± 0.95). Male athletes (4.01 ± 1.06) reported higher amotivation (*t* (630) = 4.10, *p* < 0.05) than female athletes (3.75 ± 1.09).

The independent t test for task orientation revealed significant differences on the age group (*t* (630) = 1.94, *p* < 0.05). The 16 to 18 year old age group (4.13 ± 0.44) demonstrated significantly higher task orientation than the 13 to 15 year old age group (4.06 ± 0.48). Male athletes (3.11 ± 0.69) reported significantly higher ego orientation (*t* (630) = 2.48, *p* < 0.05) than female athletes (2.98 ± 0.67). In addition, rural athletes (3.08 ± 0.69) reported significantly higher ego orientation (*t* (630) = 2.48, *p* < 0.05) than urban athletes (2.96 ± 0.65).

### Relationship between achievement goal orientation, intrinsic motivation, extrinsic motivation and amotivation

The relationship between achievement goals, intrinsic motivation, extrinsic motivation and amotivation were also explored (Table 2). Task orientation positively related to ego orientation (*r* = 0.29, *p* < 0.01). Task orientation had a significant moderate positive relationship to intrinsic (*r* = 0.55, *p* < 0.01) and extrinsic motivation (*r* = 0.39, *p* < 0.01), but a weak relationship to amotivation (r = 0.10, *p* < 0.01). Ego orientation was significantly related to intrinsic (*r* = 0.30, *p* < 0.01), extrinsic (*r* = 0.36, *p* < 0.01) and amotivation (*r* = 0.36, *p* < 0.01).

The multiple regression was conducted using the enter method. As shown in Table 3, the adjusted R^2^ presents that the contribution of the three motivation types (intrinsic, extrinsic, amotivation) explain 30.5% of the variances in task orientation. The Beta weights show that, of the three variables in the model, intrinsic motivation had the strongest influence on task orientation with a value of 0.63 (*p* = 0.001). The Beta weights for extrinsic motivation and amotivation suggested that they had no influence on task orientation. The contribution of the three motivation variables to the adjusted R^2^ for ego orientation explains 17.7% of the variances. The Beta weights shows that of the three variables in the model, extrinsic motivation and amotivation had the strongest influence on ego orientation with values of 2.05 and 2.43 respectively.

## Discussion

This study showed that older adolescent athletes were more task-oriented than younger adolescent athletes. The findings contradict previous studies (Nicholls, 1989; Xiang and Lee, 1998; Xiang et al., 2004) and Nicholls’ developmental perspective where younger children were more task-oriented, whereas older children were more ego-oriented. In terms of age differences, there could be several explanations for the present results. One explanation could be the endorsement of a task goal orientation which is related to ability. Children above the age of 12 years are more likely to use the differentiated concept of ability and children below the age of 12 years are likely to hold an undifferentiated concept of ability (Nicholls, 1989; Xiang and Lee, 1998). The emergence of task goal orientation among the older age group is encouraging as task-oriented individuals would use the less differentiated concept of ability in which ability can be developed with effort and is judged in a self-referenced manner (Xiang and Lee, 1998). One reason for this could be because older athletes show signs of maturity that focus on long term goals where success is determined by their amount of effort and time invested in sport. In order to achieve long term success, they must strive on hard work regardless of ability, prevail further in their performance and persist over time.

Another possible explanation for the observed difference in task goal orientation between the two age groups in the present study could be the presence of a task-oriented motivational climate that can influence individuals/athletes to endorse and increase the level of task-oriented goals. If a task-oriented motivational climate is promoted by emphasizing self-referenced perceptions of ability, individuals can improve their mastery by practicing hard. An individual who is self-referenced and feels successful in track and field when learning further to observed improvements is competent, autonomous and related to the sport environment.

We found that male athletes were more ego-oriented than female athletes. This is consistent with previous findings (Duda, 1989; Li et al., 1996) which showed that males tend to be more ego-oriented and females tend to be more task-oriented. The present findings suggest that male athletes’ main concern was to perform better by comparing themselves with other athletes in terms of their abilities in the competitive sports environment.

It has been proposed that socialization in the sports domain encourages male athletes to participate in competitive sports in order to develop masculine aspects of their self-identity, whereas females are often discouraged from participating in competitive sports for fear of “masculinizing” their physiques, attitudes and behaviors (Koca et al., 2005). Gender stereotyping in athletics might influence the athlete’s goal orientations with higher estimates on the gender-appropriate task whether it is masculine- or feminine-type tasks. Male athletes tend to believe that athletics promotes competition and being successful in it will lead to greater social recognition and popularity among peers as star athletes.

We found a significant difference in ego orientation between urban and rural athletes. Previous studies have not examined goal orientation based on locality. Rural athletes showed higher ego orientation than urban athletes. One possible reason for this is that rural athletes do not have the same opportunities as urban athletes in terms of competition. This is due to barriers such as their geographical isolation and financial constraints which limit the athletes’ participation in local competitions (Office for Recreation and Sport, 2007).

The findings of this study showed that there was a significant difference in intrinsic, extrinsic and amotivation between male and female athletes. Male athletes were found to have significantly higher levels of intrinsic and extrinsic motivation and amotivation. Urban athletes also showed higher intrinsic motivation than rural athletes.

Male athletes who are intrinsically motivated could have found participation in athletics interesting, enjoyable and satisfying which led them to be more intrinsically motivated than female athletes. Other studies (Ambrose and Horn, 2000; Biddle and Armstrong, 1992) have also found that male athletes displayed higher intrinsic motivation. Researchers have reported that male athletes found training in a variety of physical activities challenging, interesting and rewarding in the learning of new skills and techniques. The value and enjoyment of athletics through the accomplishment of the tasks needed to become skilled in athletics would have likely led to the feelings of competence, ability, mastery and autonomy in male athletes (Ntoumanis, 2001). Male athletes who enjoyed athletics have higher perceived competence and are more likely to make continued engagement in track and field. Therefore, it is necessary to improve perceived competence among female athletes through a wide range of activities suited to their athletic abilities. This would likely produce a greater level of intrinsic motivation (Cairney et al., 2012).

We found that male athletes reported higher levels of extrinsic motivation to participate in competitions than were reported by females. Previous studies found that males tended to display a less self-determined motivational profile than females (Ntoumanis, 2001; Pelletier et al., 1995). Deci et al. (1981) and Vallerand (1997) showed that competition can decrease intrinsic motivation and promote extrinsic motivation. This could be due to the normative comparison and outcomes that can induce male athletes to be extrinsically motivated.

In addition, male athletes could have been motivated by external factors which tend to be associated with the social environment. Male athletes tend to place importance on external factors in which their performance would be rewarded with monetary incentives, privileges, medals, recognition, material gains, chance to travel, social approval, self-worth and praise from others. These external factors can undermine intrinsic motivation.

Amotivation was found to be higher in male athletes than in female ones. This could be due to the imposition of extrinsic constraints and contingencies which are based on performance and outcome from significant subjects such as coaches, parents, and teachers who prepare athletes for competition where winning and the outcome of sporting events is paramount. Naylor (2006) stated that successful outcomes of games, influx of competitive opportunities and monetary rewards are the main ways in which athletes validate their efforts and see themselves in a positive light. This shapes their coaching behaviours and decision making during athletes’ adolescent developmental years. Therefore, the performance-related environment which is controlling and demanding in which male athletes are expected to meet would lead to them feeling amotivated. Male athletes who struggle to meet expectations that are either imposed by themselves or other significant subjects may likely diminish their self-determination. These have led to the decrease in their perceived competence which is necessary for them to be more self-determined. Ryan and Deci (2000) stated that increases in perceived competence must be accompained by a sense of autonomy in order for the enhanced feelings of competence to result in increased intrinsic motivation. Based on this premise, it is essential that action is taken to remedy the current situation in case the amotivated male athletes eventually drop out of competition.

The findings in this study also showed a significant difference in intrinsic motivation among urban and rural athletes. Urban athletes were found to be more intrinsically motivated. No significant difference was found between the groups in extrinsic motivation or amotivation.

This finding is consistent with Côté et al.’s (2006) study which revealed that the majority of elite athletes came from urban areas. Their study revealed that athletes who have access to resources such as superior facilities, were more intrinsically motivated compared to their counterparts training in rural areas where facilities were lacking or less equipped. As urban elite athletes received more support, supervision and coaching, there is a gap between athletes from urban and rural areas. Rural athletes face obstacles such as lack of facilities, limited financial resources, and inadequate, outdated and substandard training equipment (Gauthier et al., 2005). In addition, lack of access to services, for example distance, lack of transport, cost of transport and limited mobility could lead rural athletes to have a lower level of intrinsic motivation as compared to urban athletes.

Therefore, these studies conclusively showed that urban athletes either at the elite or developmental levels are more privileged as they have access to the best training, facilities, funding and services. Significant subjects such as supportive coaches and parents who demonstrate strong intrinsic desire in the development of athletes, inspire them to pursue athletic excellence (Gauthier et al., 2005). Further evidence showed that urban teacher-coaches who volunteer to support and coach the athletes despite other school obligations and family commitment are task-oriented and intrinsically motivated by intrinsic factors such as skill development, excitement/challenge, team and fun which are important in sustaining urban athletes’ participation.

The lack of facilities in rural schools could hamper efforts to inculcate interest in sports among children and coaches in rural areas. Therefore, the lack of ample opportunities in the rural areas has resulted in rural athletes having less intrinsic motivation than urban athletes.

In accordance with previous data (Duda et al., 1995; Kim et al., 2003; Ntoumanis, 2001), these findings have shown a significant relationship between goal orientations and types of motivation. Task orientation is related to intrinsic motivation, whereas ego orientation is related to extrinsic motivation. Task oriented athletes who have benefited from a task mastery environment would experience a form of satisfaction through participation in athletics because task goal orientation and higher form of self-determination share a direct relationship. The results further supported Duda’s (1992) findings that task oriented athletes were more likely to have a stronger need for competence than ego oriented individuals who possess less control over their perceptions of competence. The self-determination theory holds that intrinsic motivation is a consequence of a need to feel both competent and self-determined. It predicts a close relationship between perceived competence and intrinsic motivation in that the more competent individuals feel about performing an activity the higher their intrinsic motivation levels (Weiss and Ferrer-Caja, 2002).

The results of this study provide additional evidence about gender, age group and locality differences in adolescent’s goal orientation and types of motivation. There is a necessity in the importance of de-emphasizing an ego-oriented achievement perspective to reduce ego-orientation among males, younger and rural athletes. In addition, it would minimize the extrinsic motivation among adolescent athletes and adopt a self-determined motivation in their involvement in athletics due to the inherent pleasure in the activity itself and help to maximize their motivation to excel themselves in athletics.

## Figures and Tables

**Table 1:** Independent t-test for task orientation, ego orientation, intrinsic motivation, extrinsic motivation and amotivation as a function of age group, locality and gender

Variable	Age group		Locality		Gender	
	13–15 yr	16–18 yr	Urban	Rural	Male	Female
Task orientation	4.06 (0.48)^*^	4.13 (0.44)	4.14 (0.48)	4.06 (0.46)	4.10 (0.47)	4.06 (0.46)
Ego orientation	3.04 (0.68)	3.07 (0.70)	2.96 (0.65)^*^	3.08 (0.70)	3.11 (0.69)*	2.98 (0.67)
Intrinsic motivation	4.56 (0.94)	4.65 (0.97)	5.05 (0.94)^*^	4.83 (0.89)	5.01 (0.87)^*^	4.72 (0.92)
Extrinsic motivation	4.56 (0.94)	4.65 (0.97)	4.69 (0.91)	4.56 (0.96)	4.82 (0.89)^*^	4.32 (0.95)
Amotivation	3.87 (1.09)	3.95 (1.06)	3.84 (1.01)	3.91 (1.10)	4.01 (1.06)^*^	3.75 (1.09)

* *p < 0.05. Standard deviations appear in parentheses below means*.

**Table 2:** Correlation coefficients between task orientation, ego orientation, intrinsic motivation, extrinsic motivation and amotivation

Subscale	1	2	3	4	5
1. Task orientation	──	0.29^**^	0.55^**^	0.39^**^	0.10^**^
2. Ego orientation		──	0.30^**^	0.36^**^	0.36^**^
3. Intrinsic motivation			──	0.79^**^	0.31^**^
4. Extrinsic motivation				──	0.45^**^
5. Amotivation					──

** *p* < 0.01

**Table 3:** Multiple regression for task orientation, ego orientation, intrinsic motivation, extrinsic motivation, and amotivation

Variable		*β*	R^2^adj	*F*	*p*
Task Orientation	Intrinsic Motivation	0.63	0.305	93.34	0.000^*^
	Extrinsic Motivation	− 0.82			0.153
	Amotivation	− 0.55			0.140

Ego Orientation	Intrinsic Motivation	0.65	0.177	46.22	0.000^*^
	Extrinsic Motivation	2.05			0.268
	Amotivation	2.43			0.000

* *p* < 0.01
